# Can the Identity of a Behavior Setting Be Perceived Through Patterns of Joint Action? An Investigation of Place Perception

**DOI:** 10.3390/bs4040371

**Published:** 2014-10-13

**Authors:** Harry Heft, Justine Hoch, Trent Edmunds, Jillian Weeks

**Affiliations:** 1 Department of Psychology, Denison University, Granville, OH 43023, USA; E-Mail: weeks_j1@denison.edu; 2 Department of Psychology, New York University, New York, NY 10003, USA; E-Mail: justine.e.hoch@gmail.com; 3 Information Technology Services, Denison University, Granville, OH 43023, USA; E-Mail: Edmunds@denison.edu

**Keywords:** behavior settings, place perception, ecological psychology, structure from motion, family resemblance

## Abstract

“Behavior settings” are generated by joint actions of individuals in conjunction with the milieu features (or affordances) that are available. The reported research explores the hypothesis that the identity or meaning of a behavior setting can be perceived by means of the patterns of action collectively generated by the setting’s participants. A set of computer animations was created based on detailed observation of activities in everyday settings. Three experiments were conducted to assess whether perceivers could extract “structure from motion” (in this case, collective actions) that was specific to the particular behavior setting displayed by way of the animations. Two experiments assessed whether individuals could accurately perceive the identity of the behavior settings with such displays, and a third experiment indirectly examined this possibility by evaluating whether setting possibilities and constraints were recognized. The results offered some support for the hypothesis, and suggested several refinements in how to conceptualize a typology of behavior settings. An ecological approach to place perception is also discussed.

## 1. Introduction

After having spent more than a decade investigating children’s behavior in the laboratory [1], Roger Barker set out in the late 1940s to catalog children’s activities in more common everyday settings [2]. He was prompted to begin this laborious project after coming to the realization that in spite of his familiarity with how children behave in specific, controlled laboratory investigations, he knew little about children’s daily lives in their homes and communities. It soon became apparent to him that this state of affairs characterized the field of psychology more generally. Unlike the other sciences, psychology has neglected to develop a systematic understanding of the frequency and distribution of its most essential subject matter, behavior. He pointed out, e.g., that whereas biologists know how common or rare particular flora and fauna are, and how they are distributed in nature, psychologists have little way of knowing whether some psychological phenomenon studied in the laboratory is commonplace or atypical, and under what everyday circumstances it occurs.

In the course of this observational work, Barker hoped to understand the basis for the apparent order in children’s everyday behavior. Gradually over the first 5 or 6 years, children exhibit behavior that, by and large, is increasingly consistent with the expectations of the adult community. How can we begin to understand the basis for these fairly regular patterns of action that are produced *in situ*? In their efforts to answer this question, Barker and his collaborators [3,4] made a momentous, and yet still under-appreciated discovery about the ecological structures of everyday life.

Psychological tradition led Barker initially to make two assumptions at the outset of his attempt to find the causes of everyday actions. First, he assumed quite reasonably that the basis for order in everyday behavior was to be discovered solely at the level of individual action. After all, psychology’s focus has long been, and continues to be, on the individual, and in turn, on the ways in which happenings in the environment affect individual action and thought. The second assumption followed directly from the first: It was assumed that much of the time the specific individual actions that were observed could be tied to antecedent events stemming from other individuals and directed toward the child, such as directives from a parent, teacher, or peer. Under the broad influence of stimulus-response (S-R) thinking in psychology (or, in later decades, of input-output formulations of cognitive psychology), it seems logical to assume that actions can be tied to immediately antecedent conditions. However, data collected over a number of years by Barker’s research team indicate that both of these assumptions are questionable.

Their observational data indicated that antecedent individual occurrences (“social inputs”) were, at best, rather weak predictors of a child’s actions. Typically, fewer than 50% of child-directed actions or utterances were followed by behavior congruent with those “inputs”. This unexpected finding forced Barker to reexamine the considerable body of evidence that had been gathered and the prior assumptions that he had made. After all, there was order to children’s actions. In classrooms, children usually acted as students should; in the town’s stores, children generally behaved in ways that were appropriate to those establishments; in religious services children acted in socially normative ways for the setting, and so on. And yet, typically only in a minority of instances were these behaviors initiated by a social input from another individual. How then were these regular patterns to be explained? In an imaginative leap, Barker saw that focusing solely on the actions of an individual (the first assumption above) was inadequate to account for the data. He recognized that the best predictor of behavior was “where” in the community the child was at any particular time.

### 1.1. The Discovery of Behavior Settings and Their Components

Barker was initially led to this conclusion by recognizing that the actions of an individual child had greater variability across different locations in the community than did the actions of *different children within the same location*. This pattern indicated that something quite powerful psychologically was tied to place. However, this observation is strikingly out of step with psychological tradition in that it runs against the view that the best level of analysis for understanding individual action is at the scale of the individual (much less at more molecular levels). Indeed, Barker found that a child’s actions at any particular moment were most accurately predicted by knowing “where” she was (e.g., a music lesson, a game of dodge ball, a church service) rather than knowing details about individual child. From this starting point, efforts to understand what “where” means in psychological terms led Barker to the discovery of extra-individual, dynamic structures that he called “behavior settings”.

Not infrequently, the most surprising discoveries are hidden in plain sight by the stance one adopts. In spite of their rather “late” discovery in psychology, behavior settings have always been ubiquitous in human environments. After all, the actions of an individual are never free-standing, as if an individual is ever positioned in some empty Cartesian space—although that would seem to be how psychology has long operated. Instead, *action* is always situated. Behaviors always occur somewhere, in some context. Notably, a significant feature of the context is a dynamic, yet stable pattern of actions generated by joint participation of two or more individuals with the functional support of affordances (“milieu”). Barker called these ecological structures behavior settings. Consider, for example, an on-going class session. The setting “class session” is constituted by the patterned action of students and teachers, with the support of affordances, such as places to sit and surfaces on which to write. That is, if some number of students sit at their desks quietly, raising their hands when they wish to speak, speaking after the teacher calls on them, recording information in notebooks placed on their desks, *etc.*, then collectively their actions in conjunction with material features of that setting generate a higher-order pattern of behavior that is conducive to teaching and learning. (Of course, there are multiple patterns of action that are suitable to teaching-learning, this being only the most traditional.)

It is revealing to note that each individual’s actions are “double-sided”. On the one hand, by operating in a manner that is socially normative with respect to e.g. classroom settings, each individual directly contributes to the constitution of that setting. On the other hand, in order to be a participant in a classroom setting, the individual must limit his or her actions in ways that are socially normative in relation to such settings. In other words, individuals’ actions simultaneously contribute to the creation and maintenance of a setting and are constrained by virtue of their participation in the setting. By acting in ways that help to constitute a particular behavior setting, individuals as a matter of course limit their actions in a manner that helps to sustain that setting, even as they may be seeking their own ends within the setting.

Such reciprocal processes, whereby structured actions create social structures that constrain subsequent actions have come to be seen as commonplace by social theorists such as Giddens [5] and Bourdieu [6]. Psychologists, by remaining focused solely on individual action, are prone to miss the role of these collective eco-behavioral structures. Moreover, the occurrence of higher-order patterns that are generated by their components, and concurrently constrain the operational possibilities of those components, are typical of many natural systems [7,8].

The defining attribute of any behavior setting—that is, what distinguishes a behavior setting from a mere collection of individuals, or from a location without any individuals (e.g., an empty classroom)—is a requisite degree of *interdependence* among its components, the participants and the milieu. Barker [9] (p. 41) writes in this respect, “the phenomena of psychology and the environments in which (they occur) are interrelated; they are interdependent in the way a part of a system and a whole system are interdependent”. Establishing the degree of interdependence is an empirical matter determined by assessing the degree to which alterations in one part of a setting reverberate in other parts of the setting (for details see [3]).

Barker and his colleagues investigated behavior settings and their operations extensively over many decades [10,11]. Still, there is an untested assumption that underlies this perspective. For individuals to participate appropriately in an on-going setting, they must first be able to perceive the type of setting that they are entering. In other words, they must recognize the identity (or meaning) of that setting. This presupposition is critical for obvious reasons: if an individual cannot recognize the type of setting she is about to enter—for example, whether it is a classroom setting, or a religious service, or a team game—how is she to know what to do, and what not to do, when joining it as a participant?

Of course, this understanding must stem from a prior history of engagement in the various behavior settings one encounters within his or her community. No doubt one important facet of early social development involves being engaged as a participant in a variety of settings. Through such experiences, the individual develops a repertoire of actions-in-context. Indeed, Barker has delineated the ways in which the range of experiences, as well as the level of responsibility in a setting’s operations, covary with age [4] (pp. 116–118).

To be a participant in a behavior setting, and in doing so, to contribute to its operation, the individual must select the range of permissible behaviors that are appropriate for the setting; and such selection is contingent on perceiving the setting type. (Barker employed the term “behavior setting genotype” to refer to different settings that shared common functions and operations. For example, two different fire stations in a town would share a common genotype.) Moreover, to maintain the necessary operations that constitute a setting, individuals must be able to perceive when collective actions are beginning to deviate beyond a normative range and to threaten the viability of the setting’s purposes.

Barker was well aware of this fundamental premise about the perceived meaning of a setting. He writes, “The identification of a setting rests upon the direct perception of a synomorphic (*i.e.*, mutually supportive) relation between a standing pattern of behavior and (its) milieu” [4] (p. 53). He does offer some evidence for this possibility by appealing to the agreement among his research team in their efforts to identify existing behaviors settings. However, these individuals likely had prior familiarity with the concept of a behavior setting. Alternatively, does the documented existence of behavior settings not stand as prima facie evidence that their identity is perceivable? That does seem reasonable, but it remains to be demonstrated more directly that individuals in the course of their daily activities can recognize the identity of familiar behavior settings. Moreover, and more critically, assuming that individuals can perceive the identity of a familiar behavior setting, what is the nature of the stimulus information that specifies a particular behavior setting from the standpoint of a perceiver? These two questions—can the identity of a behavior setting be perceived, and if so, what is the nature of the information specifying its identity—are the primary concerns of the research to be reported here.

Generally speaking, there are two likely sources of information that specify the identity of a behavior setting. The most obvious source of information is the physical layout of the setting, such as its furnishings and their arrangement. Although this information is apt to be the most salient, it is not necessarily reliable. Physical layouts can sometimes be utilized for uncharacteristic purposes. A wedding can occur in an open area of a park, a raffle can occur in a church, and a classroom can be utilized for a play performance. These examples indicate that when patterns of action seem to be in conflict with physical layout, the former usually overrides the latter. Admittedly, these circumstances are atypical, but still such examples indicate that the physical layout plays mostly a supportive role in (and in some cases a hindrance to) the social dynamics of a behavior setting.

These considerations suggest that the most distinctive and reliable perceptual information specifying the meaning of a behavior setting is likely to be the collective action pattern of the individuals who contribute to its constitution. Barker posited that a behavior setting is principally the quasi-stable “standing pattern” of behavior among its participants. For this reason, our goal in this investigation was to assess whether individuals can perceive the identity of a behavior setting based *solely* on the pattern of collective actions by its participants. Admittedly, the pattern of actions is shaped by milieu possibility and constraints, but in the present work the milieu structure was not perceivable, only being at best implied through actions. We predicted that the dynamic structure that is revealed among the joint actions of setting participants over time could function as perceptual information that specifies the meaning of a familiar behavior setting.

### 1.2. Methodological Approach

To explore these issues, we adapted a methodology that was initially developed by Johansson [12,13] in the domain of event perception. Specifically, he was interested in the visual perception of motion stemming from both inanimate and animate sources. To illustrate his approach with a comparatively simple case, consider a rigid rod rotating around an axis in the field of view at 45 degrees from the fronto-parallel plane (*i.e*., an oblique rotation). He hypothesized that this object in motion would be perceived with reference to invariant geometric relations in an otherwise changing pattern of visual information. The methodology that he devised for testing this hypothesis, a *point light display*, involved placing lights at both ends of a rod, and then filming the rod in darkness with only the lighted ends of the rod visible as it rotated. Even with this minimal kinematic information, namely, two moving points of light, a rotating rod oriented at 45 degrees was readily perceived, and this is because the relative distance between the lights was preserved (invariant) in relation to the perceived angle of rotation. Specifically, as the rod (unilluminated but for the ends) rotated around a fixed axis, and end-to-end away from the viewer (projectively in the picture plane), the relative distance of the two lighted end points of the lights remained invariant. Indeed, they specify the perceived angle of rotation out of the fronto-parallel plane. Research using such point light displays has generated an extensive research literature on what has come to be called the perception of structure from motion.

Johansson [12,13] applied this same methodology to the study of animate motion by attaching lights to the joints of a moving person, and filming various actions in otherwise total darkness. He found that perceivers were readily able to identify actions such as walking, dancing, bicycling, climbing a ladder, and doing pushups, based solely on the kinematic patterns of the moving lights. Remarkably, the action could often be identified within less than a second of exposure. Later research demonstrated that other qualities of the individual are also readily perceivable, such as their gender and the weight of an unseen lifted object [14].

Runeson [14,15] described these visual displays as offering a “kinematic specification of dynamics” or in short, the KSD-principle. In the context of biological motion, Runeson [15] (p. 386) describes the KSD-principle in the context of person perception studies in the following manner: “properties pertaining to a person that have a dynamic (“causal”) role in the generation in his or her movements are specified by the resulting kinematic patterns”. In other words, he is proposing that the meaning or purpose of the action, that is, the individual’s intentions, is specified in the perceived kinetic patterns.

Those familiar with Gibson’s ecological approach to perceiving will note a strong similarity between it and Johansson’s analysis. In spite of some differences between the Johansson and the Gibsonian research programs concerning how to best analyze such motion patterns [16,17], there is agreement that perceiving involves detecting lawful regularities in a changing visual array. Both also agree that these regularities are detected by the perceptual system without the mediation of non-perceptual processes. In other words, the available visual information considered over time is sufficient to account for what is perceived. For this reason, from the standpoint of both approaches, perception of the event is taken to be direct. (Discussion of the theoretical and philosophical implications of this point goes beyond the bounds of this paper, but see [18,19].)

Applying this reasoning and methodology to our research question, we examined whether behavior setting type could be recognized with reference to the pattern of joint or collective actions among participants in a setting. The experimental materials developed for the present project were modeled after Johansson’s methodology. Instead of applying points of light to joints of the body, we represented the person schematically as a whole (see below). Our goal was to examine whether observers could identity a familiar behavior setting type from a kinematic display of actions among those involved in the setting. Because our focus was on the kinematic display of information, fixed, physical features of a setting, such as furnishings, were not included in the displays. Our assumption was that specific patterns of collective action that emerge from the operation of a behavior setting can serve to specify to a perceiver the type of setting that it is.

### 1.3. The Primary Research Materials

One member of the research team (the second author) observed and recorded the actions of participants in four public settings in a small Midwestern town (population approx. 4000). Observations were carried out over a 30 min period of time when there was a relatively high level of activity in each setting. In some cases, such times were easy to determine, as in the case of lunchtime at a small restaurant. In other cases, such as a bank lobby, several visits were necessary in order to find a time when there was considerable activity on-going.

The observer developed detailed written records of the activities of participants in each setting by noting on prepared, properly scaled floor plans the paths of locomotion of individuals through each setting and the locations where they remained in a stationary position, either standing or sitting. The estimated length of time it took for individuals to walk from one point to another in the settings was also noted on the floor plans. The public settings were the lobby of a bank, a reading room in a community library, a small restaurant that serves mostly lunches, and an ice cream shop. A fifth setting was also included, namely, a portion of an organized basketball practice that existed on videotape.

**Figure 1:**
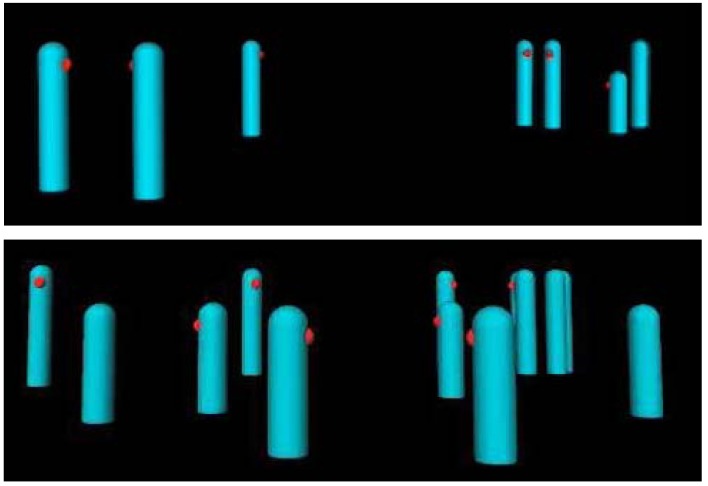
Still screen shots (cropped) of two of the setting animations. Note that in the absence of movement, the identity of these settings is nearly impossible to discern.

Working from the detailed transcribed records, as well as the one video recording, computer animations of the activities in these settings were generated using Autodesk^®^ Maya^®^ 3D animation software. The animations were created through the collaboration of the setting observer and a computer graphic designer (the third author) in order to capture the dynamics of the setting*.* Each of the animations displayed only the actions of the participants in the setting against a black field, while omitting all layout or design features (e.g., tables, chairs, walls). Still screen shots from two of the displays are presented in Figure 1.

Each behavior-setting participant was represented in the display by a blue cylinder, with a red dot on its surface marking the direction the individual was looking. Seated participants in the behavior setting were represented by a cylinder 2/3rds the height of a moving or standing one. If a participant sat or stood up during the simulated time interval, the cylinder shrank or become elongated, respectively. The only other action represented in the displays was movement (*i.e.*, displacement of the cylinders) within the setting.

Initially, each animation was based on the ten-minute interval during the observation period when activity was greatest. However, because asking participants in the subsequent experiments to view five displays, each ten minutes long, was anticipated as excessively demanding, and also because in some of the settings, several minutes transpired during the ten-minute interval when relatively little activity occurred, we further edited the animations to three-minute displays. In the case of those settings in which activity was relatively continuous, specifically, the restaurant, the ice cream shop, and the basketball game, a three-minute interval that seemed representative of the initial ten-minute animation was selected. In the case of the library reading room and the bank lobby, short intervals of time when no activity was occurring were edited out. In the end, each animation consisted of three minutes of somewhat continuous action based on the original observational records.

One obvious quality of the still screen shots in Figure 1 is noteworthy to point out. As static representations, the identity of the setting each still shot captured in a “frozen” second is indeterminate. This quality generally holds for all of the investigations that utilized the Johansson point-light display technique. When the point-light displays (and in the present case, the setting patterns) are presented dynamically, the indeterminateness is greatly reduced. In the case of the point-light displays of human movement, as noted above, clarity occurs in under a second. The research presented here assesses whether a setting’s identity can also be recognized in kinetic displays of joint action.

## 2. Experiments

Three experiments were conducted. Experiments one and two were designed to determine if observers could perceive the identity of a behavior setting based on the collective pattern of actions exhibited by participants in a behavior setting. In experiment three, observers’ were asked to indicate the degree to which certain activities would be appropriate or not in the settings as presented through the kinematic displays. This approach was intended to place the emphasis on possibilities and constraints on action rather than the explicit identification of the settings as such.

### 2.1. Experiment 1

The initial experiment employed a *ranking procedure* to evaluate how accurately participants could recognize the identity of each setting based solely on the kinematic display of information. It was intended to be a qualitative assessment of participants’ accuracy in perceiving the settings’ identity based on detecting “structure from motion”.

#### 2.1.1. Method

##### 2.1.1.1. Materials

Two types of research materials were created for use in the experiment to be reported. Computer animations of a set of target settings were produced, and a list of the names of comparison settings was generated as a way to gauge participants’ evaluation of the computer animations.

##### 2.1.1.2. Target Settings

The computer-designed animations of the restaurant, the ice cream shop, basketball game, the library reading room and the bank lobby, as described above, were employed. Each animation was three minutes in duration.

##### 2.1.1.3. Comparison Settings (Setting Names)

For each target setting, a set of four names of comparison settings was chosen to be utilized in the experiment. These four comparison settings for each target setting name were derived from an initial pre-test.

In the pre-test, participants (n = 15) rated each of twenty setting names in terms of its degree of similarity to the target setting names (see Table 1). For the purposes of generating these comparison settings, the names of the target settings (rather than the animations) were employed. Participants rated each of the twenty settings on a 1–9 scale, with 1 = “completely different pattern of activity”, and 9 = “nearly identical pattern of activity, just a few minor differences”. The order of the twenty setting names was counterbalanced across participants, as was the order of the target names.

**Table 1:** The list of the 20 settings employed in the pre-test ratings.

A list of 20 settings	
Waiting room in doctor’s office	Grocery store
Playground	Art gallery
Baseball game	Bookstore
Dining hall	Airport lounge area
Airport ticketing area	Football game
Standard church service	Poker card game
Fast food restaurant (interior)	Family holiday party
Restaurant with tables	Ticket window at a stadium
Skating rink	Standard classroom
Volleyball game	Coffee shop (like Starbucks)

Note: Similarity ratings to the name of the target settings were obtained.

Mean ratings for each of the twenty setting items in comparison to each of the five target-setting names were calculated. Based on these ratings, the setting name receiving the highest mean rating, the one receiving a mean intermediate rating, and the two settings receiving the lowest mean ratings, were selected for each target name. The four comparison setting names selected for each target setting and their mean ratings are listed in Table 2. Because pre-test participants were making these comparisons based on setting names, the name of the target setting was not included among the 20 options. However, the target setting names are in Table 2 in parentheses for the sake of clarity.

**Table 2:** The comparison setting names selected in relation to five target names for use in Experiments 1 & 2.

Rank	Bank	Library	Ice Cream Shop	Restaurant	Basketball game
1	(bank)	(library)	(ice cream shop)	(restaurant)	(basketballgame)
2	airport (7.05)	bookstore (7.35)	fast food (7.45)	coffee shop (7.6)	playground (8.67)
3	fast food (4.90)	airport (5.75)	doctor’s office (7.30)	dining hall (6.75)	volleyball game (5.67)
4	classroom (3.40)	restaurant (3.15)	dining hall (4.93)	bookstore (3.35)	dining hall (5.43)
5	playground (1.50)	Family party(2.30)	airport (3.09)	football game (1.80)	bookstore (3.66)

Note: Mean rating score (1 = completely different pattern of activity, and 9 = nearly identical pattern of activity, just a few minor differences) noted in the parentheses. (Refer to Table 1 for complete item names and the text for details).

##### 2.1.1.4. Participants

Twenty-five individuals agreed to participate in the initial investigation in exchange for course credit. The sample consisted of both male and female students who were enrolled in introductory psychology classes at an undergraduate liberal arts college.

#### 2.1.2. Procedure

Participants were told initially that they would be shown a series of computer simulations of individuals acting in some everyday settings. They were instructed that after viewing each simulation, they should examine the five comparison setting names on their response sheet and to *rank order* the comparison setting names as to how well each resembled the setting presented in the visual simulation. These five comparison settings were those selected in the pre-test, as well as the name of the setting displayed in the simulation (see Table 2). The presentation order of the animations and the orders of the comparison settings for ranking were counterbalanced across participants.

#### 2.1.3. Results and Discussion

Table 3 displays the five target settings, the average assigned rankings (1–5) of each of the five comparison settings, and the original ranking based on the pre-test (in parentheses). As can be seen, in every instance the comparison name that was a match for the simulation received either a ranking of one or two. The order of the Rankings 3, 4, and 5 derived from the pre-test were duplicated here. In short, the rankings of the setting names in relation to the target settings animations were remarkably consistent with the ranks derived from the pre-test results.

**Table 3:** The mean rankings of each comparison setting (names) relative to the target setting (animations).

Rank	Bank	Library	Ice Cream Shop	Restaurant	Basketball game
1	airport (2)	bookstore (2)	ice cream shop (1)	coffee shop (2)	basketball game (1)
2	Bank (1)	library (1)	fast food (2)	restaurant (1)	playground (2)
3	fast food (3)	airport (3)	doctor’s office (3)	dining hall (3)	volleyball game (3)
4	classroom (4)	restaurant (4)	dining hall (4)	bookstore (4)	dining hall (4)
5	playground (5)	family party(5)	airport (5)	football game (5)	bookstore (5)

Note: The pre-test ranking for each item is in parenthesis

These findings indicate that when participants viewed the target setting animations, their rankings of the comparison items for similarity to the visual animations mirrored to a large extent the rankings established from the pre-test evaluation using only setting names. That is, the comparison settings (names) were matched to kinetic setting displays (animations) with an impressive degree of accuracy.

### 2.2. Experiment 2

In order to make a more fine-grained evaluation of how accurately participants perceive the identity of the settings based on kinematic information, a quantitative investigation was designed. This second experiment was conducted along the lines of the previous qualitative ranking study, but in this case, participants were asked to assign a *similarity rating* to each comparison setting name in relation to the visual animations. Such quantitative ratings permitted more systematic comparisons among judgments.

#### 2.2.1. Method

##### 2.2.1.1. Participants

Twenty-two individuals, both male and female, participated in this experiment. They were enrolled in introductory psychology classes at an undergraduate liberal arts college and received course credit for their participation.

##### 2.2.1.2. Procedure

Participants were shown a series of brief computer animations of individuals engaging in activity in specific everyday settings (identical to those used in Experiment 1). After viewing each animation, they were given a list of five setting names and asked to rate each named setting with respect to how dissimilar (1) or similar (7) it was to the setting depicted in the animation. The five comparison setting names for each display were the same as employed in Experiment 1 (Table 3). The presentation order of the simulations and the orders of the comparison settings were counterbalanced across participants.

For each target animation, a one-way ANOVA for repeated measures was applied to the ratings of the five comparison settings. In the case of a main effect, *post hoc* comparisons (Fisher’s least significant difference (LSD)) were carried out.

#### 2.2.2. Results and Discussion

Although the degree of correspondence between comparison ratings to target settings varied across the particular target setting being evaluated, overall the patterns of the ratings were relatively accurate. Moreover, these differences in relative accuracy across the target settings are themselves quite informative (see Section 3.1). It will be useful to consider each target setting separately, beginning with those that were most readily identified (see Table 4).

A. Basketball game: (F = (4, 21) 64.13, *p* > 0.01) Participants were most accurate in recognizing this setting and distinguishing it from the options. The choice basketball game was given the highest similarity rating, and *post hoc* tests indicated that ratings for the name basketball game were significantly different from all of the other setting ratings (see Table 4). Further, the playground rating, which received the second highest rating, was significantly larger than the three remaining settings. This choice is consistent with the likelihood that participants correctly identified this setting because basketball games commonly occur on playgrounds. The third choice was another rule-governed sport, a volleyball game, and although its mean rating was low, it was significantly higher than for the bookstore. The dining hall and bookstore were given very low ratings and were not differentiated in terms of their similarity to the kinematic display.

B. Library reading room: (F = (4, 21) 8.82, *p* < 0.01) Participants most often identified this display as a library room (5.00), distinguishing it to a significant degree from all other possibilities. Still, the absolute value of the rating indicates some uncertainty based on the kinematic display. The ratings of the next three setting names (bookstore, airport lounge, and family holiday) were not significantly different from one another, but were rated as being significantly higher than restaurant.

C. Small restaurant: (F = (4, 21) 33.66, *p* < 0.01) Participants judged that it was equally likely that the patterns of activity in this kinematic display could have been that of a small restaurant, a dining hall, and a coffee shop. However, the ratings for bookstore and football game were rated as being significantly lower from that block of three, as well as from each other. What mostly accounts for the sizeable significant main effect is the comparatively low rating for football game.

D. Ice cream shop: (F = (4, 21) 2.39, *p* = 0.06) The overall pattern for this setting was quite similar to the preceding, although the F value in this case only approached statistical significance. What accounts for only a marginally significant main effect here is that the lowest ratings were not as disparate from all of the others as was the case in the previous set of judgments.

E. The bank lobby: (F = (4, 21) 12.77, *p* < 0.01) The similarity ratings for four of the settings—bank, airport lounge, playground, fast food restaurant—were not significantly different from each other. Moreover, all of them were moderate in magnitude, indicating that participants had some difficulty identifying the bank animation. What accounts for the significant F value is that classroom (1.97) was evaluated as a very unlikely possibility compared to all others, and probably it was the movement among the setting participants that ruled out the classroom.

**Table 4:** Similarity ratings (1–7) for comparison settings relative to target settings (display).

**Basketball game**	basketball	playground	volleyball	dining hall	bookstore	F value
	6.22	4.34	2.47	1.81	1.47	F = 64.13 **
	6.22 > 4.34 **, 2.47 **, 1.81 **, 1.47 **; 4.34>2.47 *, 1.81 *, 1.47 *; 2.47 > 1.47 *					
**Library reading room**	library	bookstore	airport	family holiday	restaurant	
	5.00	4.50	4.13	3.69	2.53	F = 8.82 **
	5.00 > 4.50 *, 4.13 *, 3.69 *, 2.53 **; 3.69 > 2.53 *					
**Small restaurant**	Small restaurant	dining hall	coffee shop	bookstore	football	
	5.47	4.94	4.81	3.22	1.91	F = 33.66 **
	5.47, 4.94, 4.81 > 3.22 *; 5.47, 4.94, 4.81 > 1.91 **; 3.22 > 1.91 *					
**Ice Cream Shop**	ice cream	fast food	airport	dining hall	waiting room	
	5.75	5.72	4.83	3.81	2.97	F = 2.39 n.s
**Bank**	bank	airport	playground	fast food	classroom	
	4.50	4.41	3.56	3.34	1.97	F = 12.77 **
	4.50, 4.41, 3.56, 3.34 > 1.97 **					

Note: F values for ANOVAs w/repeated measures: post hoc comparisons (*p* < 0.05 = *, *p* < 0.01 = **).

To summarize, in Experiment 2 participants correctly identified one of the five target animations among the setting options, while identifying for three animations (and one of these marginally) a “family” of setting types (see below). They did have considerable difficulty identifying one of the target animation settings (*i.e.*, the bank). In short, the similarity ratings indicated that in four of the five animations, participants identified the setting depicted in the animation or a common setting type.

### 2.3. Experiment 3

Whereas the first two experiments involved asking participants to consider the identity of the setting displayed in the kinematic animations, this third experiment focused on the degree to which various *activities* are judged to be appropriate for each setting animation. It should be noted that indicating which activities might be appropriate is a less stringent criterion than identifying the setting displayed. And yet it is a reasonable question to pose because with few exceptions a range of actions is normatively permissible in most settings. To offer an example, whereas having a meal is the canonical activity in a restaurant, other activities such as having a conversation or reading a book could fall within the bounds of appropriate behavior in most cases. Employing verbal labels as references to settings rather than perceptual displays, Price and Bouffard [20] found that individuals do understand that settings impose constraints on possible actions. Indeed, perhaps a better indication than explicitly identifying the name of a setting is in terms of the range of activities that were considered to be normatively appropriate and those judged to be beyond the normative boundaries of each setting. Of course, making such a judgment necessitates in the first place the identification of the displayed setting, and yet this need not be an explicit identification, but rather one made in the course of action and in relation to activities. In other words, when encountering an on-going behavior setting in the course of action, one may merely join into the activities without necessarily identifying the setting in an explicit manner (e.g., “This is setting X therefore I must participate thus and so”). Knowledge of setting meaning may be more tacit than that, understood in relation to on-going action possibilities and constraints (See Section 3.2).

#### 2.3.1. Method

##### 2.3.1.1. Pre-test: Comparison Activities

A pre-test of activities (n = 15) to be including in the rating portion of the experiment was conducted along the same lines as that employed in the first two experiments. Participants (n = 16) indicated on a 9-point scale (1—not appropriate; 9—very appropriate) how appropriate each of 20 named activities would be for each setting. The order of the twenty activity names was counterbalanced across participants, as was the order of the setting names.

Based on these ratings, the two activities receiving the highest mean rating, the one receiving a mean intermediate rating, and the two activities receiving the lowest mean ratings, were selected for the later assessment of each setting. The five activities selected for each target setting and their mean ratings are listed in Table 5.

**Table 5:** The activities to be assessed in relation to each animation in Experiment 3.

Bank	Library reading room	Ice Cream Shop	Restaurant	Basketball game
Depositing a check (9.00)	Reading (7.88)	Having a date (8.35)	Having a conversation (9.00)	Exercising (8.2)
Talking on the phone (6.66)	Having a conversation (5.94)	Talking on the phone (6.94)	Reading (7.08)	Having a conversation (6.52)
Having a coffee (4.88)	Talking on the phone (4.22)	Reading a book (5.11)	Writing a paper (4.75)	Dancing (4.41)
Having a meeting (2.83)	Writing a letter (3.05)	Dancing (3.23)	Playing a musical instrument (3.00)	Having a party (3.35)
Taking a nap (2.48)	Playing tennis (1.05)	Taking a nap (1.29)	Exercising (1.75)	Reading a book (1.64)

Note: These rating were based on consideration of the setting names, not the animations. Mean rating score (1 = not appropriate for the setting, and 9 = appropriate for the setting) noted in parentheses.

##### 2.3.1.2. Participants

Twenty-seven participants, both male and female, participated in this experiment. They were enrolled in introductory psychology classes at an undergraduate liberal arts college and received course credit for their participation.

#### 2.3.2. Procedure

Participants were shown the series of kinematic displays of individuals engaging in activities in specific everyday settings (identical to those used in Experiments 1 and 2). After viewing each animation, they were given a list of five *activities’ names* and asked to rate each named activity with respect to how appropriate it would be for the activity to occur in setting display, with 1 = not appropriate and 7 = very appropriate. The presentation order of the simulations and the orders of the activities for each were counterbalanced across participants.

For each target simulation, a one-way ANOVA for repeated measures was applied to the ratings of the five activities. In the case of a main effect, *post hoc* comparisons (Fisher’s LSD) were carried out.

#### 2.3.3. Results and Discussion

It bears repeating when considering the results that most settings are not highly prescriptive of the particular activities that are normatively acceptable. Exceptions are (a) those settings that have very narrow goals, prescribed procedures, and are continuously monitored by a leader, and (b) those settings sustained by mutually agreed upon rules, such as rule-governed games. Other than such instances (which even themselves allow for a measure of variability), there are varying degrees of freedom as to what is socially appropriate in most settings. Consequently, and unlike the previous experiments, our interests here primarily concerned which activities are judged to be appropriate and which not. This approach reflected the possibility that knowledge about settings may be most fundamentally grounded in a tacit understanding of action possibilities and constraints. More generally, we felt that this approach was an alternative, if more indirect way of evaluating participants’ identifying the meaning of a setting. Here again we will consider each animation in turn (see Table 6).

**Table 6:** Mean ratings of appropriateness (1–7) for each activity following the viewing of each setting animation.

**Basketball game**	having a party	dancing	having a conversation	exercising	reading a book	F values
	6.18	5.44	4.85	4.11	1.70	F = 17.99 **
	6.18, 5.44 > 4.85, 4.11, 1.70 *; 4.85 > 4.11, 1.70 *; 4.11 > 1.70 **					
**Library reading room**	talking on the phone	reading	writing a letter	having a conversation	playing tennis	
	4.74	4.07	3.78	2.70	2.63	F = 4.02, *p* = 0.056
	4.74, 4.07 > 2.70, 2.63 *					
**Small restaurant**	having a conversation	writing a paper	reading	exercising	playing a musical instrument	
	4.59	4.1	4.0	2.9	2.8	F = 15.67 **
	4.59, 4.1, 4.0 > 2.9, 2.8 *					
**Ice cream shop**	dancing	having a date	talking on the phone	reading a book	taking a nap	
	3.85	3.67	3.59	2.41	2.15	F = 4.53 *
	3.85, 3.67 > 2.41, 2.15 *; 3.59 > 2.15 *					
**Bank**	having a meeting	having coffee	depositing a check	talking on the phone	taking a nap	
	4.70	4.44	3.96	3.70	2.15	F = 4.5 *
	4.70, 4.44, 3.96, 3.70 > 2.15 *					

Note: F values for ANOVAs w/repeated measures: post hoc comparisons (*p* < 0.05 = *, *p* < 0.01 = **).

A. Basketball game (F (1, 26) = 17.99, *p* < 0.01). “Playing basketball” was not included among the activities to be rated because this setting was readily identified in the previous experiments. Eliminating this possibility allowed us to consider responses to a wider range of activities than what might have otherwise occurred. Participants rated “having a party” and “dancing” as the most appropriate activities in this animation, followed by “having a conversation and exercising”. “Reading a book” was rated as most inappropriate. Clearly participants identified the animation as displaying an activity that involved considerable movement among a group of individuals, and affordances (e.g., an open space) that would support it. “Exercising” for many in the sample is experienced as an individual activity, possibly accounting for the moderate mean rating it received.

B. Library reading room (F (1, 26) = 4.02, *p* = 0.056). In spite of the marginal F value, brief consideration of the mean differences are at least suggestive, “Talking on the phone” and “reading” were assessed as being most likely after viewing this animation, followed by “writing a letter.” Activities judged as least likely were “having a conversation” and “playing tennis”. This type of setting does, in fact, admit a wide range of action possibilities, and participants apparently perceived the animation in this vein, while ruling out other activities. Specifically, “having a conversation” (to be distinguished from solitary “talking on the phone”) and “playing tennis” were both assessed as low in appropriateness. Still, it should be noted that the means for three highest rated items were all near the mid-point of the scale, suggesting either some uncertainty about the nature of the setting, or judgment that a wide range of activities were possible, or both. That said, clearly some setting constraints were perceived.

C. Small restaurant (F (1, 26) = 15.67, *p* < 0.01). Assessment of the activities with respect to this animation followed a similar pattern to that of the previous display. The most similar items based on the pre-test results, “having a conversation”, “writing a paper”, and “reading” were significantly different in appropriateness in comparison to the two least similar activities as determined by the pre-test, “exercising” and “playing a musical instrument.” It appears that based on the animation, there was a range of activities that were judged as somewhat appropriate, and two others that were seen as outside the proper bounds. The moderate ratings here, as above, suggest a wide range of normatively appropriate actions.

D. Ice cream shop. (F (1, 26) = 4.53, *p* < 0.05) As with the basket ball game, the kinematic display of this setting was readily identified in Experiments 1 and 2 by participants, most likely because it was a familiar local place for many. For this reason, activities such as buying ice cream or waiting in line at a snack bar were excluded as options. The three activities judged to be most appropriate (“dancing”, “having a date”, “talking on the phone”) scored in the mid-range. All of these activities have a social character. Two activities fell outside a normative range (“reading a book”, “napping”).

E. Bank (F (1, 26) = 4.50, *p* < 0.05) “Having a meeting”, “having coffee”, “depositing a check” and “talking on the phone” were all assessed as moderately appropriate, while “taking a nap” was outside a range of normative action. The previous experiments showed that this setting was the most difficult to identify based on the animations, and the variability between the most highly rated activities reflect this outcome.

The primary concern of Experiment 3 was to assess whether participants could distinguish between normatively appropriate actions on the basis of the setting animations and, inversely, actions that fell outside the bounds of social normativity. We assumed that identifying setting constraints in particular would offer an alternative way for us to assess how well settings were identified based on the kinematic displays. Overall, the data support this possibility. In each instance, participants assessed a set of actions (out of five) that were likely and others that were not appropriate for the setting as perceived through the animation.

That said, it might seem surprising that in all but one case the activities receiving the highest ratings scored near the mid-point of the scale, and one of those was even below that. However, recall that in those instances where identification previously was found to be high, the most typical activity name was not included. Still, it is difficult to know if the ratings in the mid-range reflect uncertainty about a setting’s identity or recognition of a wide range of acceptable actions that might have slightly depressed all of those ratings

## 3. General Discussion

The three experiments reported here evaluated whether individuals could accurately identify a behavior setting based solely on the perceived patterns of activity exhibited among the individuals in that setting. We tested this possibility by operationalizing the question as follows: can the identity of a behavior setting be detected through “structure from motion” in a kinematic display of joint action? In Experiment 1, rankings of named comparison settings in relation to target displays were found to mirror closely the rankings determined through the initial pre-test. In Experiment 2, which employed similarity ratings, the named comparison setting that matched the target display was identified in two out of five instances, and in two other cases, it was identified as one among a small cluster of other setting possibilities. In Experiment 3, setting identity was assessed with reference judgments as to what actions are possible in the displayed settings. The aim here was to evaluate whether participants would recognize setting opportunities and constraints based solely on the animations. The results were largely consistent with that prediction. Taken collectively, the results of these experiments offer initial support for the hypothesis that individuals can perceive the identity of a behavior setting, and its affordances for action, from the pattern of joint activity among its participants. Strictly speaking, this conclusion applies only to the particular settings represented in these five animations. That said, it is assumed that setting types considered more broadly would have more or less distinctive and identifiable dynamic structures. At the same time, the equivocal character of some of the results point to a way in which the initial conceptualization of our research question can be refined.

### 3.1. Refining the Initial Formulation

One the one hand, it is not be too surprising that the identity or meaning of a behavior setting can be identified through the actions of its participants because these joint actions are among the principal constituents of the setting. That is to say, the behavior setting exists, in large part, because its participants act according to the agreed-upon norms and understood constraints of the setting. On the other hand, it was not at all obvious in advance that a setting’s identity could indeed be *perceived* through these patterns of action alone. The findings offer support for that supposition.

To be more specific, the results indicate that when individuals’ actions in a setting are constrained by more or less formalized rules that are known to its participants (e.g., as in the case of the basketball game display), onlookers have little difficulty judging the identity of the setting from the collective pattern of action specific to it. Collective actions that publically specify the identity of a setting stem from participants knowing what actions are normative in that setting, (or at least what is normative given their particular role in the setting). In other words, a setting’s identity can be conveyed by action patterns stemming from the mutually agreed-upon practices of its participants.

To a lesser degree, action patterns may also be in evidence owing to another source; namely, possibilities for actions that are solicited as well as constrained by affordances in the setting. For example, participants in the research apparently were able to differentiate the library reading room from both a bookstore and an airport waiting area based on patterns of action alone. What distinguishing patterns may have been operating here? Although the answer to this question requires further research, we speculate that a contributing factor was the relative incidence of sitting down among setting participants in each setting; and this action is only possible if the proper affordances (e.g., chairs) are available. (Recall that sitting and standing from a seated position were conveyed in the animation.) In the library animation, some individuals sat down for brief periods, while others moved about in the setting. This mix of actions would have been less common in a bookstore, which typically has few places to sit, and more common in an airport waiting area, which typically has more seating usually arranged in linear configurations. In other words, it is feasible that affordances in each setting established some possibilities and constraints for action, which in turn were reflected in the perceived kinematic patterns in the animation.

These considerations reaffirm Barker’s claim that the perceivable patterns of action that constitute a behavior setting stem principally and concurrently from two sources: one source is the set of normative actions appropriate to the setting, and the other, the affordances present in the setting. Of course, these two factors are typically co-related, as settings are designed, and in some instances selected, because their affordances support the actions of the setting. For example, individuals who are assembling to play a game of baseball will usually select a flat, open expanse, and then designate foul lines, bases, and so on. Barker [3] referred to this relationship of fit between behavior-milieu as “synomorphy.” In our view, the data suggest that differentiating one specific setting from a list of other possibilities stems from joint contributions of these two constituent properties of a behavior setting.

That said, the data also lead us to reconsider our initial conjecture that the specific pattern of collective action that emerges from the operation of a particular behavior setting narrowly specifies the type of setting that it is. In other words, our working assumption at the outset was that there would be a perceivable, prototypical pattern of activity specific to each setting type. However, prompted by the data, it would seem that this is likely to be so only in the case of behavior settings in which a restricted set of somewhat formalized rules and constraints operate. We found with the majority of our setting displays that the target animations were not identified unequivocally, but instead were judged to be one of a delimited set of possibilities. We take these findings to indicate that most settings vary in their normatively permissible actions or degrees of freedom, and for this reason, patterns of joint action in any one case may *initially* specify a “family” of behavior settings among which there is some overlap of permissible actions. That is, the patterns of action across a set of different settings sometimes share a “family resemblance” [21].

This idea of “family resemblance” among particulars stems from Wittgenstein’s [22] influential reformulation of categories. Whereas in standard accounts of category formation, particulars that belong to a common category are all assumed to share at least some feature(s) in common, the notion of sharing a family resemblance means that frequently types may be understood as belonging to a common category even though there is no one feature shared by all. For this reason, one would be hard pressed to single out explicitly the basis for their shared resemblance. And yet, a diverse set of instances is often recognized as belonging to a common family.

If we may be permitted to explore these ideas further in the interest of developing an approach to place perception, consider, for example, the behavior settings “a football match” and “a tennis match”. Both are understood to belong to the category “games” along with many other occurrences (e.g., hide and seek, card games such as poker) even though they share little in common at the level of particulars. Understanding their commonality would seem to reside at a holistic and abstract level, and awareness of the quality that they share is of a tacit rather than explicit nature. (The notion of tacit knowing is most often associated with skilled performance, including rather mundane actions, whereby an individual performs a complex task at a high level of proficiency without being able to enumerate how it was done [23].) Moreover, because the understanding that operates at this tacit, abstract level is not the type that can result from others’ pointing out to us the commonalities (because these are not readily identifiable), this kind of knowing can only come about as a result of first-hand experience in thepractices a community*.*

To illustrate the notion of family resemblance with reference to our data, the pattern of actions in the small restaurant was perceived to be as equally likely as a dining hall or a coffee shop, but clearly neither a bookstore nor a football game. Intuitively, it does seem as if these former three settings (small restaurant, dining hall, coffee shop) are of a common type, but it is difficult to specify explicitly what it is that they share in terms of particular action patterns. It may well be that this categorical understanding resides at a more abstract level of a family resemblance, along the lines of “places where one can sit and eat with others, having purchased food prepared by someone else”. Because places are not typically identified for us by others in this way, an individual’s understanding of this commonality stems from the self discovery of abstract relations rather than explicit enumeration by others. This tacit understanding comes from adopting the first person stance of a participant in such settings.

Our findings then lead us to propose that in those instances when the degrees of freedom for action are highly constrained, e.g., by formalized rules, the dynamic patterns of collective actions are sufficient to specify a particular setting type. However, more often these kinematic patterns stemming from the joint actions of participants point to a family of settings. Over time with opportunities to participate in those settings, individuals develop the expertise to differentiate the specific type of setting at hand within its family of settings [24].

In short, the research hypothesis received some support, and the results helped to illuminate a potentially richer way to approach the problem of perceiving the identity of a behavior setting. While this extended formulation awaits further empirical examination, let us consider its theoretical implications for the perception of place.

### 3.2. An Ecological Approach to Perceiving Behavior Settings

What do these considerations suggest as regards to a way of conceptualizing the process of place perception? The theoretical approach that served as the foundation for these investigations is that of ecological psychology, and more precisely, a synthesis of Gibson’s and Barker’s ecological approaches [19,25]. There are several features of an ecological approach that distinguish it from more standard approaches. Here we will consider only one of them: namely, the relationship between perceiving, conceiving, and acting.

Standard constructivist theories in psychology treat perceiving, conceiving, and acting as separate, semi-independent processes. From this stance, visual perception delivers relatively meaningless and disordered stimulus patterns to the individual. Based on these sense deliverances and drawing from a repository of stored memories, the individual employs cognition to make a subjective “best guess” (an inference or hypothesis) as to what are the most likely “external” or environmental (objective) conditions that account for this equivocal pattern. In other words, meaning or order is imposed on these sense deliverances by way of higher-order cognitive processes. As a result, experience of the environment, which is the product of an enriching non-perceptual process, is indirect (“it is in the head”). In the case of setting or place perception, psychologists have assumed for decades now that this cognitive understanding resides in mental representations of the workings of a setting in the form of a “script” [26]. In turn, the script directs the actions of the individual by way of a prescriptive algorithm. In other words, environmental perception/cognition is viewed as operating along the lines of a linear input-out process with perception, cognition, action considered to be separable, though causally connected processes, and that we experience the environment indirectly through the mediation of mental representations.

Along these same lines, Wicker [27] has described the operations of an individual in a behavior setting as a process of “sense-making”, and sees many parallels between his account and the notion of a script. Schoggen [28] was less sanguine about these parallels, and Heft [19] (pp. 269–271) has offered a critique of both approaches. In contrast, our working assumption was that kinematic patterns generated by dynamic events in the environment—in the present case, joint actions partially constituting behavior settings—are themselves perceptually meaningful, specifying the behavior setting, when these patterns are understood with respect to organism-environment transactions [24]. Their perceived meaning stems from individuals’ participating (*i.e.*, a perception-action process) in the setting, through which the distinguishing dynamic features of the setting’s operations are revealed. From this perspective, the line is blurred between perception, cognition, and action, as they are conventionally understood. Here meaningful experience is not relegated solely to cognitive processes operating independently of perception and action, but rather knowing (cognition) is integral to the perception-action process of engaging the environment. Critically, the meaning of a behavior setting is not imposed through interpretative processes operating independently of action because this meaning has its roots in the first person stance of a participant. Such a stance is that of coming to know normative action possibilities and constraints of a particular setting as a participant in it. Subsequently, the judgments from a third-person stance become possible, which was the stance the individuals in our experiments needed to adopt. The perceived meaning of a behavior setting then stems from a history of being a participant in practices of a community that include that setting. It is an understanding rooted in action. In this way, perceiving, cognizing, and acting are intertwined, inseparable, and on-going.

That said, does the evidence of equivocality in several of the perceptual judgments not weigh in favor of a constructivist account? From the latter point of view, the equivocal set of ratings indicates efforts that involve going beyond the input and coming to generating alternative hypotheses as to what the setting type is. The assumption here is that the identity of the setting must fall into one of a set of *clearly articulated* categories, and which setting it is comes about by sorting through a series of definitive (*i.e*., “clear and distinct”) hypotheses. This way of conceptualizing perception is rooted in our long tradition of Cartesian thinking wherein the grounds for certain knowledge are sought, even of a temporary nature. More recent expressions of this stance can be seen in the penchant for favoring fully specified computational models to explain perceiving and cognizing. Likewise, this approach assumes that once the setting’s identity is posited, a clearly specifiable sequence of actions is called forth. In other words, it is assumed that moments of uncertainty in perceiving and acting are *resolved* through cognition, and that certainty is imposed on these two processes through the control of top-down, computational cognitive processes.

Alternatively, if we assume that perceiving, cognizing, and acting are on-going, interlaced processes, then the perceived meaning of a pattern of dynamic events, as well as one’s course of action in light of that meaning, need not be resolved and certainty established at a fixed moments. Instead, perceiving, knowing, and acting continue to take shape “on the fly” over time and can flexibly and adaptively shift with on-going circumstances. Skilled, adaptive performance (as opposed to habitual, rote performance) is not tightly prescribed, but has a measure of “ad hoc-ness” about it as the individual continually makes fine adjustments with continuing organism-environment transactions [28].

## 4. Conclusions

In the course of daily life, we continually pass through a series of behavior settings as participants. When closely examined, these actions can be seen to be “double-sided”. On the one hand, in order to take part in the activities of a particular setting, each individual must function in ways that are consistent with the socially normative practices of that setting. On the other hand, in doing so, the individual directly contributes to the constitution and maintenance of that setting by participating in on-going collective actions. For these reasons, as Barker and his colleagues established empirically, the most revealing predictor of an individual’s actions at a particular time is knowing “where” that individual is.

The validity of this viewpoint, however, is predicated in part on the assumption that individuals are able to identify the behavior setting type that they are about to enter. Only then can they select the appropriate range of socially normative actions for that setting. If this assumption is warranted, then it must be demonstrated that the identity of a behavior setting can be perceived. Moreover, if it can be, one must be able to make a start, at least, to specify the informational basis by which a setting’s identity can be perceived. The experiments reported here provide some initial support for the view that the identity of a behavior setting can be perceived, and that its identity or meaning can be conveyed by distinctive dynamic patterns of joint action (“structure from motion”) generated by the setting’s participants. These findings provide a needed step in the development of an account of place perception.
